# Bark flammability as a fire-response trait for subalpine trees

**DOI:** 10.3389/fpls.2013.00466

**Published:** 2013-11-25

**Authors:** Thibaut Frejaville, Thomas Curt, Christopher Carcaillet

**Affiliations:** ^1^National Research Institute of Science and Technology for Environment and Agriculture – GR EMAX Mediterranean Ecosystems and RisksAix-en-Provence, France; ^2^Ecole Pratique des Hautes Etudes, Paleoenvironments and ChronoecologyParis, France; ^3^Centre for Bio-Archeology and Ecology, UMR5059, Centre National de la Recherche Scientifique, Institut de BotaniqueMontpellier, France

**Keywords:** ignitability, combustibility, top-kill, bark thickness, wood density

## Abstract

Relationships between the flammability properties of a given plant and its chances of survival after a fire still remain unknown. We hypothesize that the bark flammability of a tree reduces the potential for tree survival following surface fires, and that if tree resistance to fire is provided by a thick insulating bark, the latter must be few flammable. We test, on subalpine tree species, the relationship between the flammability of bark and its insulating ability, identifies the biological traits that determine bark flammability, and assesses their relative susceptibility to surface fires from their bark properties. The experimental set of burning properties was analyzed by Principal Component Analysis to assess the bark flammability. Bark insulating ability was expressed by the critical time to cambium kill computed from bark thickness. Log-linear regressions indicated that bark flammability varies with the bark thickness and the density of wood under bark and that the most flammable barks have poor insulating ability. Susceptibility to surface fires increases from gymnosperm to angiosperm subalpine trees. The co-dominant subalpine species *Larix decidua* (Mill.) and *Pinus cembra* (L.) exhibit large differences in both flammability and insulating ability of the bark that should partly explain their contrasted responses to fires in the past.

## INTRODUCTION

Fire is a fundamental disturbance process in terrestrial biomes acting on the ecosystem composition and functioning (Bond et al., 2005). Forecasting consequences of fire regime changes on ecosystem functioning with on-going global changes needs a comprehensive understanding of various mechanisms involved in plant response to fire (Lavorel and Garnier, 2002), particularly for ecosystems such as those at high altitude that are very sensitive to climate (Thuiller et al., 2005). Response of plant to fire differs between functional types according to biological traits (Noble and Slatyer, 1980; Lavorel et al., 1997; Pausas, 1999). The growing interest in the biological concept of flammability and its application in fire ecology address the linkages between plants and fire (Pausas and Moreira, 2012), and attempts to understand the role of fire in generating trait divergence and species persistence in fire-prone ecosystems (Keeley et al., 2011; Pausas and Schwilk, 2011). Authors argue that plant flammability properties affect the community fire behavior (Scarff and Westoby, 2006; Schwilk and Caprio, 2011) and could be under positive selection in fire-prone ecosystems (Mutch, 1970; Bond and Midgley, 1995; Pausas et al., 2012). Surprisingly, few explore the effect of flammability on the plant response to fire whereas increased flammability reduces chances of individual survival (Cohn et al., 2011). Assessing potential impacts of the plant flammability on the aboveground phytomass seems irrelevant for herbs, shrubs or saplings because these life forms are most likely to be completely burned by fire even for low-intensity fires. However, the flammability of fire-exposed tree tissues (mostly trunk bark, branches, and leaves) is of greater relevance to assess the tree susceptibility to top-kill, i.e., to endure death of the aboveground phytomass whatever the resprouting capacity.

Top-kill was commonly related to damages to stem and crown through height analyzes of burning (Van Wagner, 1973; Hély et al., 2003; McHugh and Kolb, 2003; Catry et al., 2010). While many identified surrogates of fire damage to explain the probability of top-kill (e.g., Fernandes et al., 2008), the underlying functional processes still remain to be elucidated for the emergence of a general comprehensive mechanistic model of fire-induced top-kill (Michaletz and Johnson, 2008), as well as the definition of traits involved in tree response to fire (Brando et al., 2012). Physiological explanations of post-fire stem mortality relate to the extent of thermal degradation of living tissues involved in hydraulic conductance, i.e., phloem (carbohydrate transport function), xylem (water and nutrient uptake) and cambium (source of phloem and xylem; Rundel, 1973; Michaletz et al., 2012), although cambium necrosis have been suggested as the main surrogate (Michaletz and Johnson, 2007). Bark thickness is an adaptive trait in a wide range of fire prone ecosystems (Jackson et al., 1999; Keeley et al., 2011) and was shown to be the primary determinant of cambial resistance to fire injury (Harmon, 1984; van Mantgem and Schwartz, 2003; Lawes et al., 2011) and thereby of tree top-kill (Dickinson and Johnson, 2001). Assessing the susceptibility to top-kill of tree species that dominated surface-fire prone subalpine forests of the Alps (e.g., Genries et al., 2009b) is critical facing an increasing fire risk (Schumacher and Bugmann, 2006) due to global warming (Im et al., 2010) and fuel build-up following land-use abandonment (Chauchard et al., 2010; Zumbrunnen et al., 2011).

**FIGURE 1:**
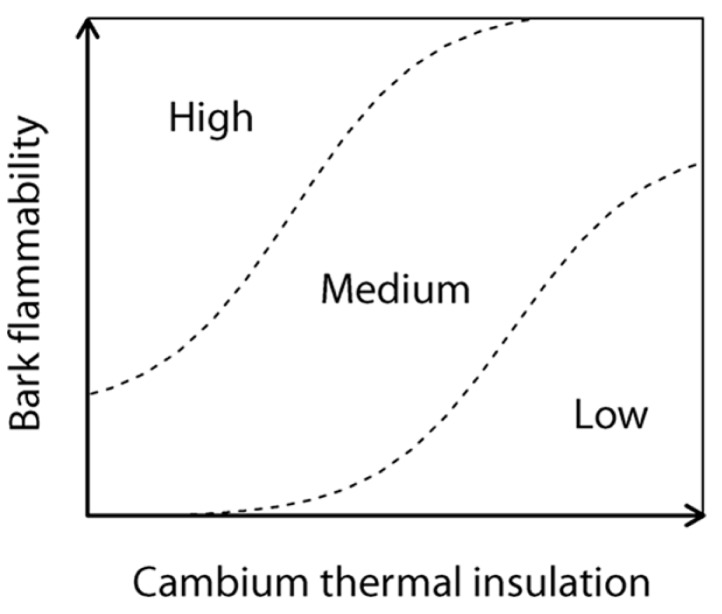
**Tree susceptibility to fire-induced top-kill (conceptual model); the cambium insulating ability of tree (as a surrogate of the insulating ability of vital conducting tissues) and the propensity of the bark to burn and char, i.e., the bark flammability, express both the tree susceptibility to endure lethal fire injuries**.

We assume that a flammable bark should increase the fire severity on inner vascular tissues and especially when bark is not deep enough to provide to trees the protection against the surrounding radiant heat. Specifically bark flammability may promote an increase in the likelihood of vascular cambium necrosis and then rely to tree response to fire by enhancing the probability of top-kill, or in a lesser extent by reducing the photosynthetic activity (Ducrey et al., 1996). To explore how and on what basis bark flammability vary depending on species and bark thickness, we performed flammability tests on eight dominant subalpine tree species of Alpine ecosystems. Flammability tests were carried out on dried samples of the bole outermost surface of trees for the sake of standardization, i.e., to remove differences in moisture that could have been induced by environmental variability between sites during sampling, and because of rehydrating bark and wood indifferently would lead to experimental bias. Even if these laboratory tests do not actually mimic field conditions (Fernandes and Cruz, 2012), flammability is a process whose variability is controlled by biological traits that can be assessed making standardized experiments (Pausas and Moreira, 2012). So we aim to test (1) the relations between bark flammability and bark insulating ability, i.e., bark thickness, and (2) to identify what bark and wood traits tie with bark flammability under low moisture conditions, i.e., when wildfires are most likely to occur and affect the trees. Finally, we aim (3) to rank the subalpine trees susceptibility to surface fires according to their bark properties. We hypothesize that bark thickness and flammability are two interrelated determinants of a fire susceptibility syndrome (**Figure 1**), i.e., thick barks must be few flammable to provide fire resistance to trees.

## MATERIALS AND METHODS

### SAMPLING

Subalpine communities are dominated by gymnosperm trees, e.g., larch (*Larix decidua* Mill.), Arolla pine (*Pinus cembra* L.), mountain pine (*Pinus uncinata* Mill.), spruce (*Picea abies* Karst.) and fir (*Abies alba* Mill.). The main associated angiosperm trees in terms of occurrence and biomass are *Betula pendula* Roth. (silver birch), *Salix caprea* L. (goat willow) and *Sorbus aucuparia* L. (rowan). These eight species were sampled in the Maurienne valley (Savoy, French Alps) – one of the driest area of the Alps – from situations with similar ecological contexts, viz. north-facing slopes at altitudes between 1900 and 2000 m a.s.l.

Bark flammability parameters were quantified for 80 trees by performing burning tests from samples of the trunk outermost surface (i.e., bark over sapwood). Ten trunks per species, in the diameter-class 7–10 cm, were sampled at ~50 cm height, the height where fire-induced injuries are likely to occur in these surface-fire prone ecosystems (Genries et al., 2009b). Logs were stored for 6 months away from moisture, to allow natural air-drying without altering the physico-chemical properties of bark and wood. Samples for burning tests were extracted from the peripheral parts of logs (outer bark, phloem, and sapwood) using a circular saw to maximize standardization. Specifically the bark surface exposed to heat and the inner wood volume were the same for all samples (3 × 2 cm area of bark and 1.5 cm sapwood depth in radial section). Differences in dry mass between samples mirror differences in wood density (WD).

### BIOLOGICAL TRAITS

For each log, three bark traits were measured from samples for burning tests while WD was measured from supplementary samples of sapwood cut under bark. Wood density (WD, g × cm^-3^) is defined as the ratio given by the oven-dried mass of a wood sample divided by its volume. Volume measurements were obtained from the geometrical dimensions of the wood core (Chave et al., 2006). The bark traits: bark thickness (BT, mm), bark roughness and proportion of outer bark (rhyt idome) over entire bark, were measured with a WinDendro 2009 device (© Regent Instrument, Québec) from all samples for burning tests. BT was estimated from the maximum value of 10 measurements per sample. In order to obtain quantitative estimates of bark fissure-depth and degree of bark roughness for a given BT (i.e., the bark thickness variability as proportion of the bark thickness), bark roughness was estimated for each sample as follows:

(1) $$y=\frac{\max\,\;(BT)-\min\,\;(BT)}{\max\,\;(BT)}\,\;$$

The bark insulating ability is given by the critical time to cambium kill which was computed from BT. The time for kill the cambium is directly proportional to the bark thickness squared (Hare, 1965; Vines, 1968; Peterson and Ryan, 1986; Hengst and Dawson, 1994; Lawes et al., 2011). We choose the simplified formula of Peterson and Ryan (1986):

(2) $$\tau \,c=2.9\,B\,T^{2},$$

where the critical time for cambium kill (minutes), τ_c_, is calculated from bark thickness, BT (cm).

### FLAMMABILITY TESTS AND PARAMETERS

Samples were dried in an oven for 72 h at 30°C to ensure gentle and uniform drying and to prevent peeling of bark. Samples were randomly selected for burning. The 80 flammability tests (10 per species) were carried out in a fume hood using a constant radiant heat from an epiradiator (reference UNE 23729-90-IR, the bark side of each sample was uniformly exposed to 215 ± 6°C, mean ± SD) with a thermocouple and a digital scale connected to data-loggers (measures of temperature and sample mass with a time resolution of 2 s). The relative amount of heat released was approximated with as the temperature recorded by a thermocouple 1 cm above the sample. Quantifying flammability properties of the aboveground outermost surface of trees required to focus on bark ignitability and on the depth of combustion from bark to inner sapwood. A burning experiment thus began when the sample was exposed to the heat source, and was considered complete after a standard time interval (180 s) to take into account only the first times of bark and wood combustion than can occur in subalpine Alpine ecosystems during slow-moving surface fires (Genries et al., 2009b). At the end of the experience, all samples were still burning. For each flammability test, the ignition delay was noted and the bark temperature at ignition point (ignition temperature) was recorded. The **Table 1** provides the set of experimental variables used to describe bark flammability. The heating rate, the heat release, the burning, and mass loss rates were computed on the entire experiment duration (flameless pyrolysis stage followed by flaming combustion stage). The burning rate and the rate of mass loss were also computed on the time period restricted to the flaming combustion period in order to assess the consumption ability of the bark regardless of its intrinsic ability to ignite.

**Table 1:** Flammability parameters, components, definitions and the processes they describe

Flammability component	Parameter	Unit	Process described
Ignitability	Ignition delay (or time to ignition)	s	Inverse fuel ability to ignite
	Ignition temperature	°C	Inverse fuel ability to ignite at low temperatures
Combustibility	(Average) heat release^†^	°C	Fuel ability to release high temperatures in the first times of heat exposure
	Heating rate (ratio of maximum temperature over its arrival time)^†^	°C × s^-1^	Fuel ability to reach high temperatures
Consumability	Rate of mass loss^†^^‡^	g × s^-1^	Speed and intensity of early thermal degradation of biomass
	Burning rate^†^^‡^	s^-1^	Speed of early fuel consumption

*Parameters are associated with the different flammability components *sensu*Anderson (1970) and Martin et al. (1994) as described by White and Zipperer (2010).*

† Variables computed on time period encompassing bark flameless pyrolysis and bark flaming combustion stages, i.e., the ignition ability (180 s from heat exposure).

‡ Variables computed also on time period of bark flaming combustion only (90 s from ignition).

### DATA ANALYSIS

Multivariate analysis of species flammability was performed by Principal Component Analysis (PCA) applied to all flammability variables. Ellipses corresponding to 95% confidence intervals (mean ± SE) were calculated, based on the average coordinates of species, as a means of representing intra-specific variation. The species position on a given axis was tested by the *v*-test (modified *t*-test, Lebart et al., 2000). Individual scores of the two first principal components of the PCA were added in a flammability index ϕ to synthesize ignition, combustion and consumption properties of subalpine tree barks, i.e., considering three major flammability components (Anderson, 1970; Martin et al., 1994). Correlations analyzes between biological traits and flammability parameters were performed using Spearman’s correlation coefficient as some variables did not exhibit a normal distribution. Simple and multiple linear regressions were conducted to test the relationship between ϕ and τ_c_, and then to evaluate the variance of bark flammability explained by biological attributes. The analyzes were performed using the *R* program (PCA from FactoMineR package, Le et al., 2008) and Statgraphics Centurion XVI © for *post hoc* Duncan tests.

## RESULTS

**FIGURE 2:**
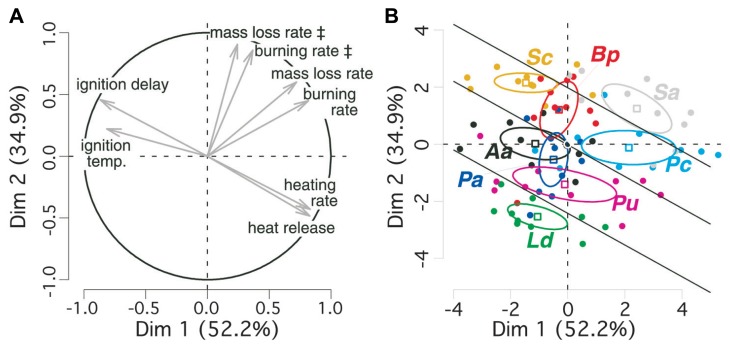
**Principal component analysis (PCA) of bark flammability for eight subalpine tree species.** 95% confidence intervals for average coordinates of species are depicted by ellipses (means ± SE). **(A)** The circle indicates correlations and contributions of flammability parameters on the two first factorial axes; **(B)** individual dispersion is depicted on the two factorial axes. The symbol **‡** indicates variables that are computed over the time period of bark flaming combustion only (90 s since ignition). Species are labeled as follow: Ld for *Larix decidua*, Aa, *Abies alba*; Pu, *Pinus uncinata*; Pa, *Picea abies*, Pc, *Pinus cembra*; Sc, *Salix caprea*; Bp, *Betula pendula*; Sa, *Sorbus aucuparia*. Bark flammability index (ϕ) was derived for all trees from the sum of their scores on the two first principal components. Significant differences (*p* = 0.05) between species were tested from ANOVA and Duncan *post hoc* tests and indicated by straight lines.

We performed a PCA for taking into account the strong colinearity among flammability parameters (Behm et al., 2004). The first factorial plane of the PCA explains almost 90% of variance in bark flammability parameters and discriminates them to different components of the flammability (**Table 1**, **Figure 2A**). Ignitability and combustibility of bark are positively expressed by the first axis. The two axes positively express bark consumability. It means that during a given period of tree exposure to heat, the earlier the bark ignition the higher the burning intensity and the stronger the bark degradation.

The *v*-test discriminated angiosperms from gymnosperms on the axis-2 (*v* > 0 for *Salix caprea*, *Betula pendula*, *Sorbus aucuparia*: *p* < 0.001; **Figure 2B**). So, the angiosperm bark is higher consumable, i.e., burned faster and lost higher biomass per time unit. *Sorbus aucuparia* and, among gymnosperms, *Pinus cembra* are located on the positive side of axis-1 (*v* = 2.42 and *v* = 2.14, respectively, *p* < 0.001; **Figure 2B**). Therefore these species have a rapidly igniting bark that burned readily reflecting a critical exposure of vascular tissues to high temperature in comparison to *Salix caprea*, *Larix deciduas*, and *Abies alba* (*v* < 0, *p* < 0.05). As ignitability, combustibility, and consumability increase with increasing scores of the two PCA axes, we added individual coordinates in the PCA plane to synthesize information of all flammability parameters into a synthetic bark flammability index noted ϕ. An inter-specific comparison of ϕ performed by ANOVA and Duncan *post hoc* test indicated four levels of significance at a 95% confidence level (*p* < 0.05, illustrated by straight lines on **Figure 2B**) from *Larix decidua* (lowest ϕ) to *Sorbus aucuparia* (highest ϕ). *Pinus cembra* bark is significantly more flammable than the other gymnosperms (*p* < 0.05). Student *t*-test indicated greater flammability for angiosperm barks (Welch modification to the degree of freedom, *t*_70_ = 5.56, *p* < 0.001).

**FIGURE 3:**
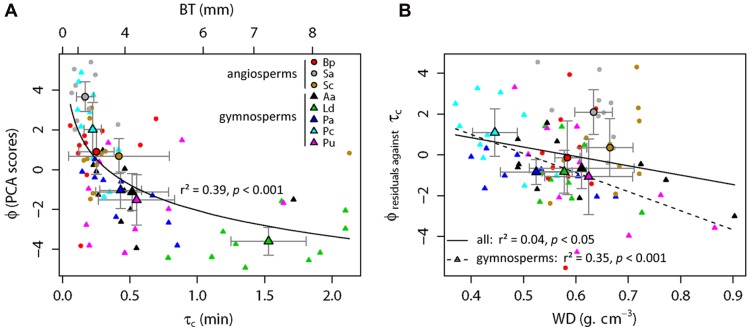
**Relative top-kill susceptibility of subalpine tree species with surface fires involving the bark flammability of subalpine trees (ϕ) *versus* the critical time for cambium kill (τ_*c*_).** This latter was computed from bark thickness (BT) using the Peterson and Ryan’s function (1986). As ϕ and τ_c_ (or BT) covaried significantly (*p* < 0.001, **A)**, regression residuals were used to explore relationship between ϕ and wood density (WD, **B**) among all trees (straight line, *p* < 0.05) and among gymnosperms (dashed line, *p* < 0.001). Species means and standard errors are indicated by larger symbols and bars, respectively.

A log-linear regression indicated a highly significant relationship (*r*^2^ = 0.39, *p* < 0.001; **Figure 3A**) between the bark flammability ϕ and the bark thickness squared that expresses the bark insulating ability. Thus distribution of trees in our fire susceptibility model (**Figures 1 and 3A**) is not random because bark flammability decreased with increasing cambial insulation, i.e., with bark thickness (BT). Specifically, bark traits (thickness, roughness and outer bark proportion) are negatively correlated with the burning rate and the mass loss rate whatever the bark ability to ignite (*p* < 0.01, **Table 2**). A variance partition analysis indicates that bark traits explains 44% of ϕ variance, BT explains 39% of the total variance, alone or throughout the interactions with outer bark proportion and bark roughness (*F*_1,78_ = 51.17, *p* < 0.001). We also investigated the role of bark density, i.e., estimated from the bark volume, the sample mass and the wood density under bark, but it did not affect significantly flammability (data not shown). After accounting for covariation between ϕ and BT, we found that bark flammability decreased with increasing wood density, especially for gymnosperms (*r*^2^ = 0.35 of residuals, *p* < 0.001, **Figure 3B**). Specifically, bark over light-wood tends to ignite earlier and at lower temperature, to burn faster and to release higher temperatures (*p* < 0.05, **Table 2**). Multiple linear regression indicated that wood density and bark thickness (both log-transformed) explained 66% of bark flammability variance of gymnosperms (*F*_1,47_ = 47.65, *p* < 0.001).

**Table 2:** Spearman correlation coefficients between the flammability parameters and the biological traits.

Flammability component	Variable	Bark thickness	Outer bark proportion	Bark roughness	Wood density	
					All (*n* = 80)	Gymnosperm (*n* = 50)
Initability	Ignition delay	<0.01	-0.22*	-0.21	0.29**	0.27
	Ignition temperature	0.10	0.02	-0.12	0.22*	0.30*
Combustibility	Heat release	<0.01	0.29**	0.20	-0.30**	-0.30*
	Heating rate	0.03	0.25*	0.21	-0.24*	-0.27
Consumability	Mass loss rate	-0.59***	-0.50***	-0.47***	0.01	-0.53***
	Mass loss rate^‡^	-0.62***	-0.64***	-0.60***	0.21	-0.35*
	Burning rate	-0.53***	-0.30**	-0.32**	-0.39***	-0.81***
	Burning rate^‡^	-0.60***	-0.49***	-0.52***	-0.15	-0.77***

The significant correlations are highlighted by gray (***p < 0.001; **p < 0.01; *p < 0.05).

‡ Variables computed over the time period of bark flaming combustion only (90 s from ignition).

The relative susceptibility of subalpine species to surface fires can be assessed from their bark properties, i.e., by their relative position in the two-dimensional theoretical model (**Figure 1**). Species located at top-left (**Figure 3A**), e.g., *Pinus cembra* and angiosperm species (*Sorbus aucuparia*, *Betula pendula*, and *Salix caprea*) should be the most susceptible to endure bole damage due to both flammable and poor insulating barks. *Larix decidua* has the lowest susceptibility to top-kill with the least flammable bark and the greater cambial insulation. Other gymnosperms (*Picea abies*, *Abies alba*, and *Pinus uncinata*) are characterized by an intermediate susceptibility due to low flammability of their bark despite short times before cambial necrosis (bottom-left location, **Figures 1 and 3A**).

## DISCUSSION

We found species-specific signatures of flammability properties among gymnosperm and angiosperm subalpine trees, i.e., their barks had varied ability to ignite and to burn. Especially, we found that bark thickness controls bark flammability and act not only as cambium insulation**.** Because inter-specific differences in flammability may relate to different parameters (Behm et al., 2004), we used a bark flammability index ϕ, which represents a set of burning properties of bark that are expected to promote an increase in the likelihood of vascular cambium necrosis during slow-moving surface fires. This first analysis of bark flammability, carried out in relation to the well-known insulating function of bark depth, provides some new features about the function of flammability in the comprehensive study of plant-fire relationships. By now, studies have focused on a non-intuitive hypothesis that enhancing flammability can be adaptive for “pyrogenic” plants (Mutch, 1970; Bond and Midgley, 1995; Schwilk and Kerr, 2002; Pausas et al., 2012). On the contrary, we hypothesized that the flammability properties of a living tree must interact with other fire-related traits to shape its potential response to fire.

### BARK FLAMMABILITY TRAITS

As expected, we found that the variance of bark flammability among trees related significantly with the main trait related to tree survival following surface fires, i.e., the bark thickness (Harmon, 1984; Michaletz and Johnson, 2008). It implies that the location of a tree species on the bivariate plane of bark flammability *versus* bark insulating ability (fire-susceptibility conceptual model, **Figures 1 and 2A**) is not random but mostly determined by the cambium insulating ability of the species itself. In other words, subalpine trees do not fulfil the top-right side of this conceptual diagram implying that trees cannot exhibit both a thick and a flammable bark. These results do not indicate that low bark flammability has the same effect that its thickness to provide tree resistance to cambium necrosis, but it highlights the hidden potential role of flammability. When the fire environment allows the bark ignition, i.e., low moisture levels, this supports the hypothesis that bark inhibits the increase of underlying cambial temperature respective to both its depth (Hare, 1965; van Mantgem and Schwartz, 2003; Lawes et al., 2011) and its related flammability. In other words, bark depth prevents both the heat transfer to inner living tissues and the severity with which the bark burns.

Among other studied bark traits, the roughness and the outer to inner thickness ratio did not explain much more flammability variance than the only bark thickness. These results corroborate findings about heat transfer rate from bark surface to the inner cambium, where roughness, density and moisture content of bark had also little effect compared to bark depth (Hengst and Dawson, 1994; Pinard and Huffman, 1997; Brando et al., 2012). Otherwise, we found that one third of bark flammability variance is explained by variation in wood density in gymnosperms, irrespective to bark thickness (**Figure 3B**). Specific features in wood structure and chemical composition among hardwood species (angiosperms) may override the strong relationships between wood density and flammability observed among softwood species (gymnosperms). Wood density controls thermal diffusivity (Papió and trabaud, 1990) affecting ignition and the rate of fuel consumption (Anderson, 1970; Nagaoka et al., 1998). For a given stem diameter, wood density may act as a heat sink that limits the bark heating, inasmuch as larger trees are more resistant to fire damage because heat sink capacity increases with tree mass (Peterson and Arbaugh, 1989). Furthermore, Brando et al. (2012) suggested that high wood density increased chance of tree survival following fires in a neotropical forest. Authors argued for indirect effects linked to tree ability to compartmentalize wood decay (Romero and Bolker, 2008). From these findings wood density appears an important trait of tree response to fire. By needing laboratory tests under controlled conditions, this depth analysis of flammability did not encompass all possible sources of variation, for instance the bark moisture content that need a specific gradual experiment. Bark thermal properties should change with increasing moisture content due to higher heat capacity. Nevertheless, as bark flammability mostly related with bark thickness, it is likely that this fire-resistance trait must keep its inhibiting effect on flammability by driving the amount of moisture that the bark may contain.

### FIRE-SUSCEPTIBILITY OF SUBALPINE TREES

Our results imply that bark flammability should decrease with tree size because the rate at which bark thickens for each species depends directly on the rate of tree diameter growth (Harmon, 1984). Thus it is likely that bark flammability could have a relevant selective meaning for small or young trees. The distribution of subalpine trees in the fire-susceptibility diagram (**Figure 1**) indicated a higher range of bark flammability among thin-barked species (left side of **Figure 3A**). This is of importance since these species would theoretically have similar cambial resistance on the basis of the bark depth *versus* τ_c_ relationships (Peterson and Ryan, 1986). Thus high bark flammability should enhance the probability of cambium necrosis for small trees and differences in bark flammability could lead to different rates of top-kill. We advocate that taking into account the bark flammability would improve our ability to predict stem mortality. In fact, this may explain why top-kill is sometimes underestimated in the literature (Jones et al., 2006; Hood et al., 2007; Lawes et al., 2011). Moreover, it has been shown that a flammable bark increases the heat transfer to the cambium due to a reduction of bark thickness and a blackening of bark surface, with consequent greater differences of fire sensitivity among species (Gill and Ashton, 1968) than expected (Vines, 1968).

The increasing fire-prone conditions in European subalpine forests needs to largely improve our knowledge about the response to fire of dominant trees. The lack of data availability on post-fire tree mortality wears down our ability to test the hypotheses and confront the findings of this study. Among gymnosperm trees, the Arolla pine has a thin bark and a light wood and thus seems the most susceptible to incur lethal damages from surface fires due to a highly flammable and low insulating bark. At the contrary, larch has the thicker and so the least flammable bark that result in the highest potential resistance to surface fires, as found in other ecosystem types (Sannikov and Goldammer, 1996; Smith and Fisher, 1997). The contrasted position of these two co-dominant subalpine species in the fire-susceptibility gradient (**Figure 3A**) is consistent with outcomes of paleobotanical studies (Genries et al., 2009a; Blarquez and Carcaillet, 2010). Indeed larch (*Larix decidua*) had an independent behavior facing fires in the past millennia whereas the higher fire-sensitive Arolla pine (*Pinus cembra*) declined when fire intervals were too short (i.e., <150 years), which prevents trees to become fire resistant, i.e., large enough to acquire both low bark flammability and high cambium insulation.

An emerging set of studies in fire ecology argued that the concept of flammability has a significant place in the underlying processes of fire-plant relationships (Pausas and Moreira, 2012). This study highlighted some new insights about the biological meanings of tree bark flammability and probable relationships with tree response to fire under low moisture conditions. Knowledge of the diversity of mechanisms responsible for fire-induced tree top-kill is needed to predict the responses of newly fire-prone ecosystems to global changes. We stress the need to study the function of flammability properties of outermost tissues of trees on their response to fire, because quantifying flammability of bark, leaves and crown architecture for several climate scenarios (low moisture levels) must improve our comprehension about species susceptibility to incur stem and crown damages.

## Conflict of Interest Statement

The authors declare that the research was conducted in the absence of any commercial or financial relationships that could be construed as a potential conflict of interest.

## AUTHOR CONTRIBUTIONS

Thibaut Frejaville, Thomas Curt, and Christopher Carcaillet conceived and designed the experiments. Thibaut Frejaville performed the experiments and analyzed the data. Thibaut Frejaville wrote the paper. Thomas Curt and Christopher Carcaillet have significantly improved the manuscript.

## References

[B1] Anderson H. E. (1970). Forest fuel ignitability. *Fire Technol.* 6 312–319 10.1007/BF02588932

[B2] Behm A. L. Duryea M. L. Long A. J. Zipperer W. C. (2004). Flammability of native understory species in pine flatwood and hardwood hammock ecosystems and implications for the wildland-urban interface. *Int. J. Wildland Fire* 13 355–365 10.1071/WF03075

[B3] Blarquez O. Carcaillet C. (2010). Fire, fuel composition and resilience threshold in subalpine ecosystem. *PLoS ONE* 5:e12480 10.1371/journal.pone.0012480 20814580 PMC2930012

[B4] Bond W. J. Midgley J. J. (1995). Kill thy neighbor – an individualistic argument for the evolution of flammability. *Oikos* 73 79–85 10.2307/3545728

[B5] Bond W. J. Woodward F. I. Midgley G. F. (2005). The global distribution of ecosystems in a world without fire. *New Phytol.* 165 525–537 10.1111/j.1469-8137.2004.01252.x 15720663

[B6] Brando P. M. Nepstad D. C. Balch J. K. Bolker B. Christman M. C. Coe M. (2012). Fire-induced tree mortality in a neotropical forest: the roles of bark traits, tree size, wood density and fire behavior. *Glob. Change Biol.* 18 630–641 10.1111/j.1365-2486.2011.02533.x

[B7] Catry F. X. Rego F. Moreira F. Fernandes P. M. Pausas J. G. (2010). Post-fire tree mortality in mixed forests of central Portugal. *For. Ecol. Manag.* 260 1184–1192 10.1016/j.foreco.2010.07.010

[B8] Chauchard S. Beihle F. Denis N. Carcaillet C. (2010). An increase in the upper tree-limit of silver fir (*Abies alba Mill.*) in the Alps since the mid-20th century: a land-use change phenomenon. *Forest Ecol. Manag*. 259 1406–1415 10.1016/j.foreco.2010.01.009

[B9] Chave J. Muller-Landau H. C. Baker T. R. Easdale T. A. Steege H. Webb C. O. (2006). Regional and phylogenetic variation of wood density across 2456 neotropical tree species. *Ecol. Appl.* 16 2356–2367 10.1890/1051-0761(2006)016[2356:RAPVOW]2.0.CO;2 17205910

[B10] Cohn J. S. Lunt I. D. Ross K. A. Bradstock R. A. (2011). How do slow-growing, fire-sensitive conifers survive in flammable eucalypt woodlands? *J. Veg. Sci.* 22 425–435 10.1111/j.1654-1103.2011.01280.x

[B11] Dickinson M. B. Johnson E. A. (2001). “Fire effects on trees,” in *Forest Fires: behavior and Ecological Effects*. eds Johnson E. A. Miyanishi K. (New York: Academic Press), 477–525

[B12] Ducrey M. Duhoux F. Huc R. Rigolot E. (1996). The ecophysiological and growth responses of Aleppo pine (*Pinus halepensis*) to controlled heating applied to the base of the trunk. *Can. J. For. Res.* 26 1366–1374 10.1139/x26-152

[B13] Fernandes P. M. Cruz M. G. (2012). Plant flammability experiments offer limited insight into vegetation–fire dynamics interactions. *New Phytol.* 194 606–609 10.1111/j.1469-8137.2012.04065.x 22288940

[B14] Fernandes P. M. Vega J. A. Jiménez E. Rigolot E. (2008). Fire resistance of European pines. *For. Ecol. Manag.* 256 246–255 10.1016/j.foreco.2008.04.032

[B15] Genries A. Mercier L. Lavoie M. Muller S. D. Radakovitch O. Carcaillet C. (2009a). The effect of fire frequency on local cembra pine populations. *Ecology* 90 476–486 10.1890/07-1740.1 19323231

[B16] Genries A. Morin X. Chauchard S. Carcaillet C. (2009b). The function of surface fires in the dynamics and structure of a formerly grazed old subalpine forest. *J. Ecol.* 97 728–741 10.1111/j.1365-2745.2009.01518.x

[B17] Gill A. Ashton D. (1968). The role of bark type in relative tolerance to fire of three central Victorian Eucalypts. *Aust. J. Bot.* 16 491–498 10.1071/BT9680491

[B18] Hare R. C. (1965). Contribution of bark to fire resistance of southern trees. *J. For.* 63 248–251

[B19] Harmon M. E. (1984). Survival of trees after low-intensity surface fires in Great Smoky Mountains National-Park. *Ecology* 65 796–802 10.2307/1938052

[B20] Hély C. Flannigan M. Bergeron Y. (2003). Modeling tree mortality following wildfire in the southeastern Canadian mixed-wood boreal forest. *For. Sci.* 49 566–576

[B21] Hengst G. E. Dawson J. O. (1994). Bark properties and fire resistance of selected tree species from the central hardwood region of North America. *Can. J. For. Res.* 24 688–696 10.1139/x94-092

[B22] Hood S. M. McHugh C. W. Ryan K. C. Reinhardt E. Smith S. L. (2007). Evaluation of a post-fire tree mortality model for western USA conifers. *Int. J. Wildland Fire* 16 679–689 10.1071/WF06122

[B23] Im E. S. Coppola E. Giorgi F. Bi X. (2010). Local effects of climate change over the Alpine region: a study with a high resolution regional climate model with a surrogate climate change scenario. *Geophys. Res. Lett.* 37 L05704 10.1029/2009GL041801

[B24] Jackson J. F. Adams D. C. Jackson U. B. (1999). Allometry of constitutive defense: a model and a comparative test with tree bark and fire regime. *Am. Nat.* 153 614–632 10.1086/303201 29585646

[B25] Jones J. L. Webb B. W. Butler B. W. Dickinson M. B. Jimenez D. Reardon J. (2006). Prediction and measurement of thermally induced cambial tissue necrosis in tree stems. *Int. J. Wildland Fire* 15 3–17 10.1071/WF05017

[B26] Keeley J. E. Pausas J. G. Rundel P. W. Bond W. J. Bradstock R. A. (2011). Fire as an evolutionary pressure shaping plant traits. *Trends Plant Sci.* 16 406–411 10.1016/j.tplants.2011.04.002 21571573

[B27] Lavorel S. Garnier E. (2002). Predicting changes in community composition and ecosystem functioning from plant traits: revisiting the Holy Grail. *Funct. Ecol.* 16 545–556 10.1046/j.1365-2435.2002.00664.x

[B28] Lavorel S. McIntyre S. Landsberg J Forbes T. D. A. (1997). Plant functional classifications: from general groups to specific groups based on response to disturbance. *Trends Ecol. Evol.* 12 474–478 10.1016/S0169-5347(97)01219-6 21238163

[B29] Lawes M. Richards A. Dathe J. Midgley J. (2011). Bark thickness determines fire resistance of selected tree species from fire-prone tropical savanna in north Australia. *Plant Ecol.* 212 2057–2069 10.1007/s11258-011-9954-7

[B30] Le S. Josse J. Husson F. (2008). FactoMineR: an R package for multivariate analysis. *J. Stat. Softw.* 25 1–18

[B31] Lebart L. Morineau A. Piron M. (2000). Statistique Exploratoire Multidimensionnelle. Paris: Dunod

[B32] Martin R. E. Gordon D. A. Gutierrez M. E. Lee D. S. Molina D. M. Schroeder R. A. (1994). “Assessing the flammability of domestic and wildland vegetation,” in *Proceedings of the 12th Conference on Fire and Forest Meteorology* (Bethesda: Society of American Foresters), 796

[B33] McHugh C. W. Kolb T. E. (2003). Ponderosa pine mortality following fire in northern Arizona. *Int. J. Wildland Fire* 12 245 10.1071/WF02054_CO

[B34] Michaletz S. T. Johnson E. A. (2007). How forest fires kill trees: a review of the fundamental biophysical processes. *Scand. J. For. Res.* 22 500–515 10.1080/02827580701803544

[B35] Michaletz S. T. Johnson E. A. (2008). A biophysical process model of tree mortality in surface fires. *Can. J. For. Res.* 38 2013–2029 10.1139/X08-024

[B36] Michaletz S. T. Johnson E. A. Tyree M. T. (2012). Moving beyond the cambium necrosis hypothesis of post-fire tree mortality: cavitation and deformation of xylem in forest fires. *New Phytol.* 194 254–263 10.1111/j.1469-8137.2011.04021.x 22276783

[B37] Mutch R. W. (1970). Wildland fires and ecosystems – A hypothesis. *Ecology* 51 1046–1051 10.2307/1933631

[B38] Nagaoka T. Kodaira A. Uehara S. (1998). “Relationship between density and the ignitability and combustibility of wood,” in *Proceedings of the Third Asia-Oceania Symposium on Fire Science and Technology*, 197–208

[B39] Noble I. R. Slatyer R. O. (1980). The use of vital attributes to predict successional changes in plant communities subject to recurrent disturbances. *Plant Ecol.* 43 5–21 10.1007/BF00121013

[B40] Papió C. Trabaud L. (1990). Structural characteristics of fuel components of five Meditarranean shrubs. *For. Ecol. Manag.* 35 249–259 10.1016/0378-1127(90)90006-W

[B41] Pausas J. G. (1999). Response of plant functional types to changes in the fire regime in Mediterranean ecosystems: a simulation approach. *J. Veg. Sci.* 10 717–722 10.2307/3237086

[B42] Pausas J. G. Alessio G. A. Moreira B. Corcobado G. (2012). Fires enhance flammability in *Ulex parviflorus*. *New Phytol.* 193 18–23 10.1111/j.1469-8137.2011.03945.x 22039968

[B43] Pausas J. G. Moreira B. (2012). Flammability as a biological concept. *New Phytol.* 194 610–613 10.1111/j.1469-8137.2012.04132.x 22489901

[B44] Pausas J. G. Schwilk D. (2011). Fire and plant evolution. *New Phytol.* 193 301–303 10.1111/j.1469-8137.2011.04010.x 22221150

[B45] Peterson D. L. Arbaugh M. J. (1989). Estimating postfire survival of Douglas-fir in the Cascade Range. *Can. J. For. Res.* 19 530–533 10.1139/x89-084

[B46] Peterson D. L. Ryan K. C. (1986). Modeling postfire conifer mortality for long-range planning. *Environ. Manag.* 10 797–808 10.1007/BF01867732

[B47] Pinard M. A. Huffman J. (1997). Fire resistance and bark properties of trees in a seasonally dry forest in eastern Bolivia. *J. Trop. Ecol.* 13 727–740 10.1017/S0266467400010890

[B48] Romero C. Bolker B. M. (2008). Effects of stem anatomical and structural traits on responses to stem damage: an experimental study in the Bolivian Amazon. *Can. J. For. Res.* 38 611–618 10.1139/X07-205

[B49] Rundel P. W. (1973). The relationship between basal fire scars and crown damage in giant sequoia. *Ecology* 54 210–213 10.2307/1934393

[B50] Sannikov S. N. Goldammer J. G. (1996). “Fire ecology of pine forests in northern Eurasia,” in *Fire of Ecosystems in Boreal Eurasia* eds Goldammer J. G. Furyaev V. V. (Dordrecht: Kluwer Academic Publishers) 151–167

[B51] Scarff F. R. Westoby M. (2006). Leaf litter flammability in some semi-arid Australian woodlands. *Funct. Ecol.* 20 745–752 10.1111/j.1365-2435.2006.01174.x

[B52] Schumacher S. Bugmann H. (2006). The relative importance of climatic effects, wildfires and management for future forest landscape dynamics in the Swiss Alps. *Glob. Change Biol.* 12 1435–1450 10.1111/j.1365-2486.2006.01188.x

[B53] Schwilk D. W. Caprio A. C. (2011). Scaling from leaf traits to fire behaviour: community composition predicts fire severity in a temperate forest. *J. Ecol.* 99 970–980 10.1111/j.1365-2745.2011.01828.x

[B54] Schwilk D. W. Kerr B. (2002). Genetic niche-hiking: an alternative explanation for the evolution of flammability. *Oikos* 99 431–442 10.1034/j.1600-0706.2002.11730.x

[B55] Smith J. K. Fisher W. C. (1997). Fire Ecology of the Forest Habitat Types of Northern Idaho. Gen. Tech. Rep. INT-GTR-363. Ogden: USDA Forest Service

[B56] Thuiller W. Lavorel S. Araújo M. B. Sykes M. T. Prentice I. C. (2005). Climate change threats to plant diversity in Europe. *Proc. Natl. Acad. Sci. USA* 102 8245–8250 10.1073/pnas.0409902102 15919825 PMC1140480

[B57] Vines R. (1968). Heat transfer through bark, and the resistance of trees to fire. *Aust. J. Bot.* 16 499–514 10.1071/BT9680499

[B58] van Mantgem P. Schwartz M. (2003). Bark heat resistance of small trees in Californian mixed conifer forests: testing some model assumptions. *For. Ecol. Manag.* 178 341–352 10.1016/S0378-1127(02)00554-6

[B59] Van Wagner C. E. (1973). Height of crown scorch in forest fires. *Can. J. For. Res.* 3 373–378 10.1139/x73-055

[B60] White R. H. Zipperer W. C. (2010). Testing and classification of individual plants for fire behaviour: plant selection for the wildland–urban interface. *Int. J. Wildland Fire* 19 213–227 10.1071/WF07128

[B61] Zumbrunnen T. Pezzatti G. B. Menéndez P. Bugmann H. Bürgi M. Conedera M. (2011). Weather and human impacts on forest fires: 100 years of fire history in two climatic regions of Switzerland. *For. Ecol. Manag*. 261 2188–2199 10.1016/j.foreco.2010.10.009

